# Secure Access Control and Large Scale Robust Representation for Online Multimedia Event Detection

**DOI:** 10.1155/2014/219732

**Published:** 2014-07-22

**Authors:** Changyu Liu, Bin Lu, Huiling Li

**Affiliations:** ^1^School of Computer Science and Engineering, South China University of Technology, Guangzhou 510006, China; ^2^School of Computer Science, Carnegie Mellon University, Pittsburgh, PA 15213, USA; ^3^School of Computer Science, Wuyi University, Jiangmen 529020, China; ^4^State Key Laboratory of Pulp and Paper Engineering, South China University of Technology, Guangzhou 510640, China

## Abstract

We developed an online multimedia event detection (MED) system. However, there are a secure access control issue and a large scale robust representation issue when we want to integrate traditional event detection algorithms into the online environment. For the first issue, we proposed a tree proxy-based and service-oriented access control (TPSAC) model based on the traditional role based access control model. Verification experiments were conducted on the CloudSim simulation platform, and the results showed that the TPSAC model is suitable for the access control of dynamic online environments. For the second issue, inspired by the object-bank scene descriptor, we proposed a 1000-object-bank (1000OBK) event descriptor. Feature vectors of the 1000OBK were extracted from response pyramids of 1000 generic object detectors which were trained on standard annotated image datasets, such as the ImageNet dataset. A spatial bag of words tiling approach was then adopted to encode these feature vectors for bridging the gap between the objects and events. Furthermore, we performed experiments in the context of event classification on the challenging TRECVID MED 2012 dataset, and the results showed that the robust 1000OBK event descriptor outperforms the state-of-the-art approaches.

## 1. Introduction

As one of the most interesting aspects of multimedia content analysis, the multimedia event detection (MED) is becoming an important research area for computer vision in recent years. According to the definition by the National Institute of Standards and Technology (NIST) [1], an event (1) is a complex activity occurring at a specific place and time, (2) involves people interacting with other people and/or objects, (3) consists of a number of human actions, processes, and activities that are loosely or tightly organized and that have significant temporal and semantic relationships to the overarching activity, and (4) is directly observable. A MED task is to indicate whether an event is occurred in a specified test clip based on a standard event kit [1], which includes an event name, a textual definition, a textual explication with an attribute list, an evidential description, and a set of illustrative video examples. Although there are many other definitions available, such as the MED definitions from the NIST, the research on the MED is still far from reaching its maturity. Most of the current researches are focused on specific areas, such as sports video [2], news video [3], and surveillance video [4]. These approaches do not perform well when used for the online or web based event detection due to two types of issues, which are the secure access control issue and the large scale robust representation issue. Thus, we developed an online multimedia event detection system, trying to provide general MED services.

The first issue is about how we can obtain a secure access control for the online multimedia event detection system. Compared with that of traditional distributed systems, it is a kind of service relationships between access control subjects and objects in the online multimedia event detection system. The service could establish, recombine, destruct, and even inherit efficiently to requested parameters which cannot be satisfied well by traditional access control models, such as the role based access control model [5], where an authorization to the subject could not change parametrically and dynamically with its environment. In order to fuse dynamically by a recombination as well as a distribution of resources and services to enhance an access control over diversified applications of our online system, this paper proposed a tree proxy-based and service-oriented access control (TPSAC) models for the online multimedia event detection system and gave performance evaluations on the cloud simulation platform CloudSim [6].

The second issue is about how to obtain a large scale robust representation for the online multimedia event detection system. In order to perform a more generic and complicated online MED, a very large amount of labeled training videos are required for training event classifier. This would become a quite challenging task, especially when there are a large number of events involved. Because one event was usually composed of several objects that meet specific relationships, one solution to the challenge is to use event descriptor models that were learned from standard annotated image datasets, such as the ImageNet challenge dataset [7], to represent the events and to use the spatial bag of words tiling approach to represent their relationships which can bridge the gap between the objects and events. In this paper, we developed a 1000-object-bank (1000OBK) event descriptor for the online MED task. Inspired by the object bank scene descriptor of Li et al. [8, 9], the 1000OBK used 1000 pretrained object detector models to form a semantically-rich representation for MED. The individual object detectors in the 1000OBK are based on a mixture of two root components that were trained by the deformable part model of Felzenszwalb et al. [10, 11]. The outputs of detectors are transformed into a multiscale feature space by the max-pooling approach to generate 1000 response pyramids. While the original object bank [8, 9] offers a rich and high-level image representation, a severe issue lies in the curse of dimensionality when we want to make an extension to include a large number of detectors for the online MED. Note that, for each object, the original object bank [8, 9] uses a 252 dimension feature vector. Thus, the dimension of the feature vector is 252000 when there are 1000 object categories in the bank. The 1000OBK solves the problem by using a mean-pooling approach to sample interest patches on response pyramids of 1000 generic object detectors to generate a 252 dimension feature vector for each interest patch. The interest patches for each frame were then encoded by a spatial bag of words tiling approach to bridge the gap between the objects and the events. In the experiment and analysis section, we show that our 1000OBK is a robust event descriptor which achieves better performances than the state-of-the-art approaches on the TRECVID MED 2012 dataset.

**Figure 1:**
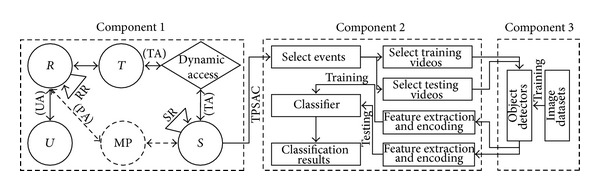
Online multimedia event detection framework.

There are three key components for our online multimedia event detection system, which are an access control component (component 1), an online event detection component (component 2), and an offline object detector training component (component 3), as shown in Figure 1. A user was firstly verified by the TPSAC model (see Section 3) in the component 1 to determine if he or she has the necessary permission to obtain an event detection service through a dynamic permission tree. If the verification is successful, the user can proceed to use the pre-trained object detectors in Component 3 to extract and encode features (see Sections 4 and 5) of selected events for training and testing the event classifier (see Section 5), either by a manual mode where the user should choose positive videos as well as negative videos in the TRECVID MED 2012 dataset for a specific event through retrievals in our system, or by an automatic mode where the system would use the default classifier that be trained previously, as shown in Component 2 of Figure 1. These object detectors of 1000OBK event descriptor were trained offline on the ImageNet challenge 2012 dataset by a mixture model of the state-of-the-art deformable part model (DPMs) object detector (see Section 4) from [10, 11], as shown in Component 3 of Figure 1. Finally, the classification results for selected events and video clips were shown graphically in our web system.

## 2. Related Work

The study on access control has always been one of the hottest areas in the field of information security. One of its key goals is to prevent unauthorized users from modifying, deleting, and revealing illegally the protected data. The traditional access control models can be classified into a matrix model, a safety classification model, an authorization rule model, and an XML model and can be also divided into a discretionary access control model, a mandatory access control model, and a role based access control (RBAC) model. By introducing a role between the subject and the privilege, the RBAC model separates logically users from their privileges and achieves greater manageability and security. Many researchers made extensive studies on the RBAC since it was firstly proposed at 1992. With the development of the service oriented technology and the grid technology, the study on access control achieved a great progress in recent years. In the service oriented aspect, the paper [12] provided a service oriented access control model to solve the safety requirement issue that the authentication and authorization mechanism of middleware could not meet the resource sharing and the service requesting between many different nodes and systems. A workflow based and services oriented role based access control (WSRBAC) model was put forward in paper [13], by introducing a role actor to ensure that the access control strategy could change rapidly with the status of workflows and services between roles and users in service-oriented architecture (SOA). In the grid technology aspect, a community authorization service (CAS) was presented to settle basically the authorization management of global as well as local grids in paper [14]. But the CAS didn't take into account the dynamic grid environment due to its static authorization mechanism [15]. In this paper, we proposed a tree proxy-based and service-oriented access control (TPSAC) model for the online multimedia event detection system.

In computer vision, the MED is becoming a hot research area. There are several key components in one MED task, which are the training and testing datasets, the raw features, the feature encoding and pooling methods, and the classification methods. Firstly, the datasets play a fundamental role in MED tasks, which include the image datasets such as Caltech 101 [16], Caltech 256 [17], MIT dataset serials [18], Label Me [19], ImageNet [7] and Pascal VOC [20], and the video datasets, such as HMDB 51 actions [21], KTH human action [22], UCF 50 [23], UCF 101 [24], UCF sports [25], hollywood human action [26], UIUC sports event [27], and TRECVID MED [1]. In this paper, we adopted the TRECVID MED 2012 dataset that is provided by the NIST. Secondly, there are four kinds of raw features, which are the visual feature, the audio feature, the text feature, and the concepts based feature. There are many popular visual features for representing multimedia contents, which include SIFT [28], color SIFT (CSIFT) [29], transformed color histogram (TCH) [30], motion SIFT (MoSIFT) [31], space-time interest point (STIP) [32], dense trajectory (DTF) [33], object bank [8, 9], and action bank [34]. Thirdly, the sparse coding [35], the fisher vector coding [36], and the spatial bag of visual words [37] are three main feature encoding and pooling approaches for representing multimedia contents. Last, there are many popular classifiers available for the MED task, such as support vector Mamhine (SVM) [38], Kernel ridge regression (KRR) [39], Kernel logistic regression (KLR) [40], adaptive boosting (AdaBoost) [41], and many modified versions of them. There are also many annual competitions for the object and event detection, such as the ImageNet challenge [7], the PASCAL VOC challenge [20], and the TRECVID workshop [1]. Supported by the NIST, the TRECVID MED task has not only attracted quickly many top research groups, such as the CMU E-Lamp research group which participated in the TRECVID MED task for the past six years and proposed many effective event detection approaches, but also became the popular testing bed for the MED.

Most of previous researches on MED have focused on detecting simple events for specific applications. Sadlier and O'Connor [42] proposed an audio-visual feature based framework for event detection in broadcast video of multiple different field sports. Zhang and Chang [43] developed a system for baseball video event detection and summarization using superimposed caption text detection and recognition. Xu et al. [44] proposed an approach for event detection from the live sports game using webcasting text and broadcast video. Li [45] proposed a probabilistic model to incorporate both content and time information in a unified framework to determine the approximate number of events from the articles count-time distribution. These researches have contributed to MED in different ways. However, no work has been put forward on how to design a generic and robust online multimedia event detection system. In this paper, we give several solutions about these.

## 3. The TPSAC Model

### 3.1. Basic Concepts

**Definition 1 (Permission Package Set, PPS).**

**Figure 2:**
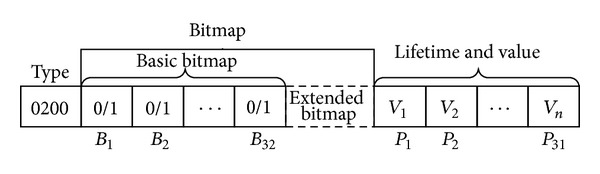
The data structure of PPS package.

The permission package is a uniform storage format for meta permission sets of various roles and a dynamic permission attribute of the proxy-based tree* AccBTree*. The data structure of PPS package consists of three sequential parts, as shown in Figure 2. The first part acts as a type identifier. The second part denotes a permission bitmap. The third part is a value sequence, which contains a weight and a lifetime, of the meta permission set which is in the same order with that of the second part bitmap.

**Definition 2 (Service, S).**

The* service* is an important concept in the SOA architecture. It is a kind of logic units, which is able to provide specific and reusable business functions to some other application systems. Loose coupling is one of the main service attributes.

**Definition 3 (Proxy-based Tree Set, T).**

The proxy-based tree* AccBTree*, which encloses the role based meta permission set, is a dynamic object that was generated when a role was activated by a user. It can be described by a quadruple 〈*R*, *PPS*, *ST*, *Lifetime*〉, where *R* is the activated role set, *ST* is the tree status set, and* Lifetime* is the tree lifetime.

**Definition 4 (Meta Permission Sets, MPS).**

Because the system resource OBS = FO ∪ DO, where FO is the function object and DO is the data object, the total meta permission set MPS = {(obj, op, *i*)∣obj ∈ OBS, op ∈ OPS, *i* ∈ *N*}, where MetaPerm = (obj, op, *i*) is a meta permission and an elementary unit during authorization, OPS is the operation set, and *I* is the lifetime or the weight that reflects the credibility and reliability of its role. Involved already the information of op and *i* in the PPS package, the meta permission set can be also expressed as MPS = {(obj, pps)∣obj ∈ OBS, pps ∈ PPS}. After getting rid of the overlapping permission, the total valid permission set *P* = {*p*∣∀ *p*_*i*_ ∈ MPS∧∀*p*_*j*_ ∈ MPS∧*i* ≠ *j* → *p*_*i*_∩*p*_*j*_ = *Ø*}.

**Definition 5 (User, Permission, and Tree Assignment).**

Let DU = {*u*∣*u* ∈ *U*}, EU = {*r*∣*r* ∈ *R*}, DP = {*p*∣*p* ∈ MPS}, *EP*⁡ = {*r*∣*r* ∈ *R*}, DT = {*t*∣*t* ∈ *T*} and ET = {*s*∣*s* ∈ *S*}. Then we define UA = {(*u*, *r*)} as user assignment relationship on DU→EU, PA = {(*p*, *r*)} as a permission assignment relationship on DP→EP, and TA = {(*t*, *s*)} as a tree assignment relationship on DT→ET.

### 3.2. Model Definitions

In order to satisfy fully the integrity constraints of task flows and enhance system security, the user permission is not only subject to the performed role but also subject to the actual task status. When a role is activated by one user, it activates accordingly a dynamic and temporary proxy-based tree* AccBTree*. As an atomic function in SOA, the* AccBTree *could be created parametrically in a task and be released after a task. Thus, the system could recycle the previous assigned user permission.

**Definition 6 (TPSAC Model).**

TPSAC = 〈*U*, *R*, MP, *T*, *S*〉, where *U* is the user set, *R* is the role set, MP is the meta permission set, *T* is the Proxy-based tree set, *S* is the service set, UA is the user assignment, PA is the permission assignment, and TA is the tree assignment. Then RR is the compound and inherited role relation, and SR is the constrained service relation. The process when a role obtains a permission service is also the one when a proxy-based tree is spanned with its status changing. The status and the constraint condition of this tree determine whether a service is allowed. There are three phases in TPSAC, which are a global static permission assignment, a proxy-based tree spanning, and a dynamic permission adjustment.

### 3.3. Model Algorithms

The TPSAC based service tree is actually a resource tree with permission information. So the TPSAC tree is a kind of multiway tree. In order to locate and create conveniently a tree, this paper adopts a child-sibling list to transform the multiway tree to a binary permission tree. The basic classes we used for access control can be defined in Algorithm 2.

In order to generate a spanning tree, a depth-first traversal algorithm was adopted recursively in the function* TAccessBTree:: CreateTree (TNode*∗*root*), where there were two steps in the spanning process of a binary permission tree (see Algorithm 1), which were a creation of a multiway permission tree and a transform from a multiway tree to a binary tree.

## 4. The 1000OBK Event Descriptor

### 4.1. The Feature Vector

Based on the original object bank [8, 9], we proposed a 1000-object-bank event descriptor, using the following steps. 

*(1) Getting 3D Interest Points by the Harris3D Corner Detector.* We used the Harris3D corner detector [32] for extracting 3D interest points from key frames of given video clips. The task of the Harris3D corner detector is to find 3D interest points which have significant changes in spatial temporal directions.

*(2) Getting the HOG Feature Pyramid.* We resized firstly the video frames so that the width is 320 pixels and the height is determined by the aspect ratio and then used six scales, which are 1, 0.707, 0.5, 0.3535, 0.25, and 0.17677, to have a further resizing on those video frames to generate an image pyramid for each frame. For HOG feature, we set the bin size to be 9 in 0°–360°, the cell type to be rectangular, the cell size to be 8 × 8 pixels, the block type to be R-HOG, the block size to be 2 × 2 cells, and the block spacing stride size to be 8 pixels.

*(3) Getting the Response Pyramid.* The state-of-the-art deformable part model (DPMs) object detector from [10, 11] was used. In our model, we used a mixture model of DPMs with two root components as our object detector. Each of these components works on 6 scales of a HOG pyramid. As a result, there are 12 response maps in the final response pyramid. 

*(4) Generating the 1000OBK Feature Vector.* We constructed our 1000OBK feature vector from responses of many object detectors on the HOG feature pyramid we obtained before. For each of the interest points that obtained by the Harris3D corner detector on the key frames, we extracted 12 response patches with each patch from one 8 × 8 dimension subwindow that located on one of the 12 response maps. Then, a three-level spatial pyramid was applied to each of the 8 × 8 dimension response patches, with 1 × 1 grid on the first level, 2 × 2 grids on the second level, and 4 × 4 grids on the third level. Each grid was sampled by the max-pooling method from its corresponding areas of the response patch. So, we got a 21 (1∗1 + 2∗2 + 4∗4) dimension feature vector for every one of the 12 response patches, resulting in a total of 12∗21 = 252 dimension vector for representing one interest patch from one object detector. A mean-pooling was then performed on each of the 252 dimension among the 1000 types of 252 dimension feature vector with each type for one object detector. Hence, the final dimension of the 1000OBK feature vector was 252, which was used to represent one patch of the interest point.

### 4.2. Object Detector Models

It can be observed from above steps that the object detector plays an important role for generating our 1000OBK event descriptor. In this paper, we used the deformable part model (DPMs) [10, 11] to train our 1000 objects detector models from the ImageNet challenge 2012 dataset, which provides the definition of 1000 objects along with 732–1300 training images, 50 validation images, and 100 test images for each of the 1000 object categories. Because there are no bounding boxes for the test images in the ImageNet challenge 2012 dataset, we split its training dataset into a training dataset where we chose 100 positive images and 400 negative images for each object category and a testing dataset where we chose randomly 1300 images for each object category. We used the trained 1000 object models as the root filters of our 1000OBK event descriptor.

## 5. Event Representation

### 5.1. Event Feature Extraction

Before feature extraction, we resized the video frames width to 320 pixels with their height to a size that determined by the aspect ratio and selected the key frames with a shot boundary detection algorithm. Specifically, the target video was firstly sampled on every 5 frames to get its color histogram and a histogram subtraction was conducted between every two consecutive sampled frames. Then, depending on whether the subtraction value was larger than a predefined threshold, a decision of whether a frame was a shot boundary was made. Last, the frame in the middle of two shot boundary frames was taken as the key frame of this shot. After the key frames selection, the Harris3D corner detector [32] was used to detect interest points for each of the selected key frames. Then feature extraction was conducted on the frame patches that were near the interest points. In order to evaluate the event detection performance by our 1000OBK event descriptor, we also extracted object bank (OBK) feature [8, 9], MoSIFT feature [31], and Kanade-Lucas-Tomasi (KLT) trajectories feature [46, 47].

### 5.2. Encoding and Classification

In order to bridge the gap between the objects and events, a spatial bag of words tiling was adopted as a feature encoding method after the raw features extraction in our experiment. For each clip, we got four kinds of feature vectors, which are 1000OBK, OBK, MoSIFT, and KLT trajectories, for the detected interest points on those key frames. Each kind of features was clustered by *k*-means algorithm. We used 50 clips for each event and 1000 clips for total 20 events, to generate four codebooks. Each of them had a size of 4096. After a generation of these codebooks, we can describe a key frame by such a 4096 dimensions feature vector. For each interest point of this key frame, we find its nearest cluster center and add one to that dimension. Normalization was performed then after the calculation for all the interest points in one key frame. We also performed a tilting approach with patterns of 1 × 1, 2 × 2 and 1 × 3, resulting in a 32768 = (1∗1 + 2∗2 + 1∗3) × 4096 dimension feature vector for each key frame.

For events classification, we used the support vector machine (SVM) classifier, since it has been widely used by several research groups for TRECVID MED and has shown its robustness [48, 49]. Given a training set {(*x*_*i*_,*y*_*i*_)}_*i*=1_^*n*^ with input data *x*_*i*_ ∈ *R* ^*n*^ and class labels *y*_*i*_ ∈ {−1,1}, the SVM tries to solve the following optimization problem:

(1) min⁡w,b,ξ⁡ 12||w||2+C∑i=1nξiSubject to: yi(wTϕ(xi)+b)≥1−ξi, ξi≥0, i=1,…,n,

where *ϕ*(*x*_*i*_) is the function for mapping *x*_*i*_ into a higher dimensional space, *C* > 0 is the penalty parameter, and *ξ*_*i*_ is the nonnegative slack variables. Then, the decision function of SVM is as follows:

(2) $$\begin{array}{c} \mathrm{sign}\left(\overset{n}{\underset{i=1}{\sum }}a_{i}y_{i}K\left(x,x_{i}\right)+b\right), \end{array}$$

where  *K*(*x*, *x*_*i*_) = *ϕ*(*x*_*i*_)^*T*^ *ϕ*(*x*_*j*_) is called the kernel function, *a*_*i*_ is the *i*th Lagrange multiplier which can be obtained by solving the dual form of the primal optimization problem, and *N*_SV_ is the number of support vectors which have *a*_*i*_ ≠ 0. For SVM, we used the exponential Chi Square (exp-*χ* ^2^) Kernel [50, 51], which is also an additive kernel. It can be defined as follows, where *γ* is a kernel controlling parameter:

(3) $$\begin{array}{c} K\left(u,v\right)=\exp\left\{-\gamma \overset{n}{\underset{i=1}{\sum }}\frac{{\left(u_{i}-v_{i}\right)}^{2}}{u_{i}+v_{i}}\right\}. \end{array}$$

## 6. Experiment and Analysis

### 6.1. Dataset

**Figure 3:**
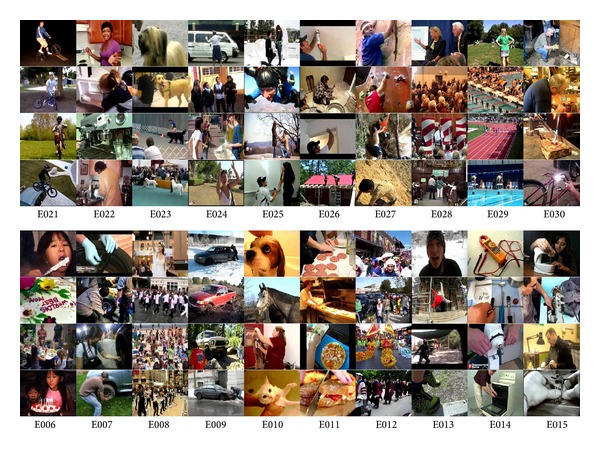
Sample frames of 20 events in TRECVID MED 2012 dataset.

We used the TRECVID MED 2012 development dataset that was provided by NIST for our experiments. The dataset had a total of 42466 clips and 20 prespecified events, which are E006: birthday party, E007: changing a vehicle tire, E008: flash mob gathering, E009: getting a vehicle unstuck, E010: grooming an animal, E011: making a sandwich, E012: parade, E013: parkour, E014: repairing an appliance, E015: working on a sewing project, E021: attempting a bike trick, E022: cleaning an appliance, E023: dog show, E024: giving directions to a location, E025: marriage proposal, E026: renovating a home, E027: rock climbing, E028: town hall meeting, E029: winning a race without a vehicle, and E030: working on a metal crafts project.  Figure 3 showed four sample frames for each of the 20 events in TRECVID MED 2012 dataset. Then, we split this dataset into a training set and a testing set. For the training set, we select randomly 20 positive clips and 100 negative clips for each of the 20 events. The other 40066 clips were used as the testing set. Then, we run our experiments three times with a different combination of positive clips as well as negative clips on the Blacklight Linux server at the Pittsburgh Supercomputing Center and reported the average results.

### 6.2. Evaluation Metrics

There are many evaluation metrics available for the MED. In our experiments three groups of them were used, which are the average precision (AP) and the mean average precision (MAP), the probability of missed detection (*P*_Miss_) and the mean probability of missed detection (MP_Miss_), and the minimum normalized detection cost (MinNDC) and the mean minimum normalized detection cost (MMinNDC). They are all the official evaluation metrics used by NIST for TRECVID MED evaluation [52, 53]. The metrics in the first group are defined in (4) and (5) separately, where *N*_*P*_ is the number of total positive clips for a specific event, *N*_tp_ is the number of true positive clips, *N*_fp_ is the number of false positive clips, *i* is for one event, *n* is the number of total events which is set to be 20 in this paper, and AP is the average precision and MAP is the mean average precision. A higher AP or MAP denotes a better performance. Consider

(4) $$\begin{array}{c} \text{AP}=\frac{1}{N_{P}}\overset{N_{P}}{\underset{N_{\text{tp}}=1}{\sum }}\frac{N_{\text{tp}}}{N_{\text{tp}}+N_{\text{fp}}} \end{array}$$

(5) $$\begin{array}{c} \text{MAP}=\frac{1}{n}\overset{n}{\underset{i=1}{\sum }}{\text{AP}}_{i}. \end{array}$$

The metrics in the second group are defined in (6) and (7) separately, where *N*_Miss_ is the number of positive clips for a specific event that is less than the detection threshold, *N*_*P*_ is the number of positive clips for a specific event, *P*_Miss_ is the probability of missed detection, and MP_Miss_ is the mean probability of missed detection. A lower *P*_Miss_ or MP_Miss_ denotes a better performance. Consider

(6) $$\begin{array}{c} P_{\text{Miss}}=\frac{N_{\text{Miss}}}{N_{P}} \end{array}$$

(7) $$\begin{array}{c} {\text{MP}}_{\text{Miss}}=\frac{1}{n}\overset{n}{\underset{i=1}{\sum }}P_{{\text{Miss}}_{i}}. \end{array}$$

The metrics in the third group are defined in (8) and (9) separately, where *C*_Miss_ = 80 is the cost for missed detection, *P*_Miss_ is the probability of missed detection,  *C*_FA_ = 1 is the cost for false alarm,  *P*_*T*_ = 0.001 is a constant defining the priori rate of event instances.  *P*_FA_ is the probability of false alarm and can be calculated by *P*_FA_ = *N*_FA_/(*N*_*T*_ − *N*_*P*_), where *N*_FA_ is the number of nonpositive clips for a specific event, *N*_*T*_ is the total number of clips,  *N*_*P*_ is the number of positive clips for a specific event, MinNDC is the minimum normalized detection cost, and MMinNDC is the mean minimum normalized detection cost. A lower MinNDC or MMinNDC denotes a better performance. Consider

(8) $$\begin{array}{c} \text{MinNDC}=\frac{C_{\text{Miss}}\times P_{\text{Miss}}\times P_{T}+C_{\text{FA}}\times P_{\text{FA}}\times \left(1-P_{T}\right)}{\min\left(C_{Miss}\times P_{T},\;C_{\text{Miss}}\times \left(1-P_{T}\right)\right)} \end{array}$$

(9) $$\begin{array}{c} \text{MMinNDC}=\frac{1}{n}\overset{n}{\underset{i=1}{\sum }}{\text{MinNDC}}_{i}. \end{array}$$

### 6.3. Evaluating the TPSAC Model

For evaluating the performance of the TPSAC model, this paper adopted a widely used cloud simulation platform CloudSim [6], which was developed by the University of Melbourne. In order to make the experimental environment to be close to the real cloud computing scene, we firstly introduced 13 federate models from paper [54]. Then, we set up a service interactive scene TPSAC simulation in the CloudSim. After that, we configured the corresponding service provider (SP) and service consumer (SC) as SP = {SCUT_CCNL_Srv1, CMU_CQ_Srv1, CMU_CQ_Srv2} and SC = {USER1, USER2, USER3, USER4, USER5} and then chose the total SP entities of 1000 with 80%, 10%, and 10%, respectively, and the total SC entities of 2000 with 50%, 20%, 10%, 10%, and 10%, respectively. All these codes were executed on an Ubuntu 12.04 LTS desktop. The other experiment configurations were similar as [55, 56]. Two experiments were conducted to evaluate the TPSAC performance, which are the impact of the bitmap length and meta permission length on the TPSAC performance and the impact of the thread pool size on the TPSAC performance.

#### 6.3.1. Varying the PPS's Bitmap Length and the Meta Permission Length

**Figure 4:**
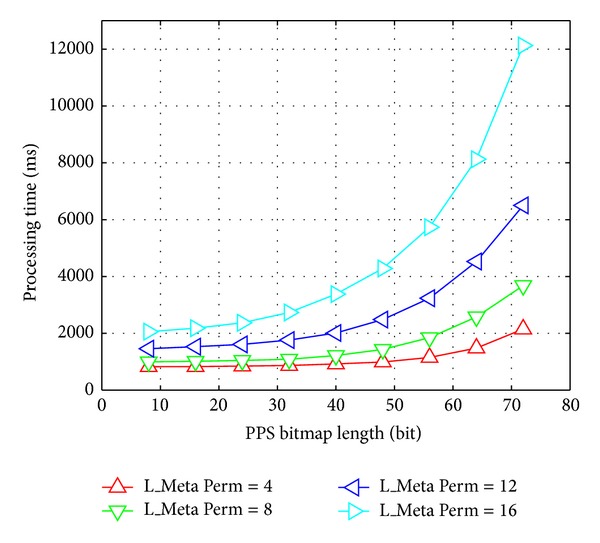
Processing time with respect to PPS bitmap length.

The goal of the first experiment was to test the relation among the TPSAC processing time *T*, the PPS bitmap length* L_Bitmap,* and the PPS meta permission length* L_MetaPerm*. Hence, we fixed the thread pool size at* L_Pool *= 32 and set* L_Bitmap *= 8∗*i*,* L_MetaPerm *= 4∗*j*, where 1 ≤ *i* ≤ 9 and 1 ≤ *j* ≤ 4. Then, we plotted the *T* value for each configuration, as shown in Figure 4.

It can be seen that (1) for the impact from the PPS bitmap length, the *T* value grew slowly when* L_Bitmap *≤ 32 and rapidly when* L_Bitmap *> 32 for all kinds of meta permission lengths and (2) for the impact from the meta permission length, the *T* value increased more sharply when* L_MetaPerm *= 4 or 8 than* L_MetaPerm *= 12 or 16. So we chose* L_Bitmap *= 32 and* L_MetaPerm *= 8.

#### 6.3.2. Varying the Thread Pool Size

**Figure 5:**
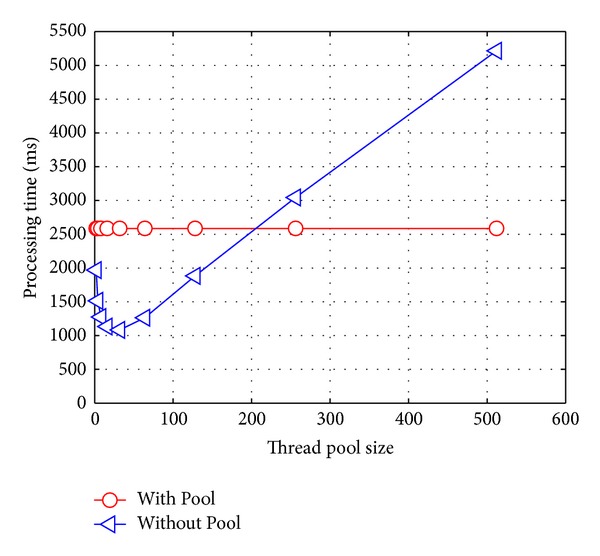
Processing time with respect to thread pool size.

The goal of the second experiment was to test the relation between the* T* value and the thread pool size* L_Pool*. Hence, we fixed* L_Bitmap *= 32,* L_MetaPerm *= 8 and set* L_Pool *= 2^*k*^, where 1 ≤ *k* ≤ 9. Then, we also plotted the *T* value for each configuration. In order to analyze conveniently the influence from using a thread pool, a *T* value without a thread pool was also plotted. The experiment results were shown in Figure 5.

It can be concluded that (1) the usage of a thread pool could improve efficiently the performance of the TPSAC server, especially when there are a large number of concurrent requests from SP and SC and (2) the change of the thread pool size has a significant impact on the specific server. If the size is too small, an access control task shall be not treated timely. Otherwise, it will lead to a larger cost for the threads synchronization and switch; that is, the optimization problem of the thread pool size should be taken into account at the beginning. So, we preset the* L_Pool* to be 32, which could then be changed with its dynamic environment.

### 6.4. Comparing 1000OBK with Baseline Approaches

We compared our 1000OBK with some of the best descriptors, which are object bank [8, 9], Kanade-Lucas-Tomasi (KLT) trajectories [46, 47], and MoSIFT [31]. For object bank [8, 9], we used the code at [57]. The object bank [8, 9] used two state-of-the-art detectors which are the latent SVM object detectors and a texture classifier by Hoiem [58] to generate a 44604 (177 (no. of objects) ∗ 12 (no. of scales) ∗(1^2^ + 2^2^ + 4^2^) (no. of grids)) feature vector for each frame. For Kanade-Lucas-Tomasi (KLT) trajectories, we used the standard KLT tracker [46, 47]. Specifically, there are about 100 interest points are detected in each frame and these points are tracked through the video clip for 15 frames, which were described by HOG, HOF and MBH. For MoSIFT, we used the implementation of paper [31] with default parameter settings. The MoSIFT descriptor combines both local appearance which is same as the original SIFT and local motion which is computed through an optical flow pyramid to extend the SIFT descriptor to the third dimension. As shown in paper [31], the MoSIFT outperformed the-state-of-the-art approaches for human action recognition in a large and complex real world surveillance dataset.

**Figure 6:**
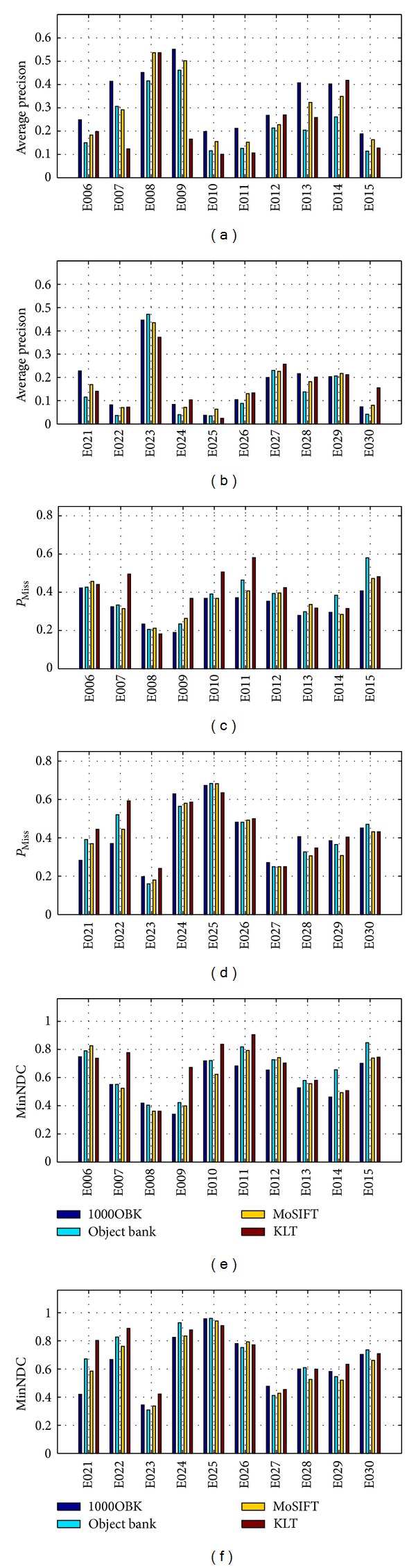
Comparison of MED performance among 1000OBK, object bank, MoSIFT, and KLT on the 20 events of TRECVID MED 2012 dataset, with (a–b) for AP, (c–d) for *P*_Miss_, and (e–f) for MinNDC.

Then, we used the LibSVM library [38] for SVM and tune the *C* from 2^−5^, 2^−3^,…, 2^15^ and the *γ* from 2^−15^, 2^−13^,…, 2^3^ under 20-fold cross validation with each fold for one of the 20 events. We ran our experiments three times with different combinations of positive clips as well as negative clips on the Blacklight Linux server at the Pittsburgh Supercomputing Center and reported the average results.  Table 1 showed a comparison of these descriptors for the average MED performance. Note that a higher MAP indicates a better performance, while a lower MP_Miss_ or MMinNDC indicates a better performance. Best results for each evaluation metric are highlighted. We can observe that our 1000OBK is the best descriptor, the MoSIFT is the second best descriptor, and the KLT trajectories or the object bank is the worst descriptor for all of the three evaluation metrics. Next, we made a MED performance comparison of these four descriptors on each of the 20 events, as shown in Figure 6. It can be seen that our 1000OBK event descriptor obtained the best performance on 10 events for average precision, on 10 events for *P*_Miss_, and on 9 events for MinNDC. Note that there are total 20 events. The comparisons here demonstrated clearly that our 1000OBK event descriptor outperforms object bank, MoSIFT, and KLT trajectories for MED.

### 6.5. Evaluating the 1000OBK Event Descriptor

In this subsection, we evaluate the robustness and the event classification performance of our 1000OBK event descriptor.

#### 6.5.1. Robustness with Respect to Training Size

**Figure 7:**
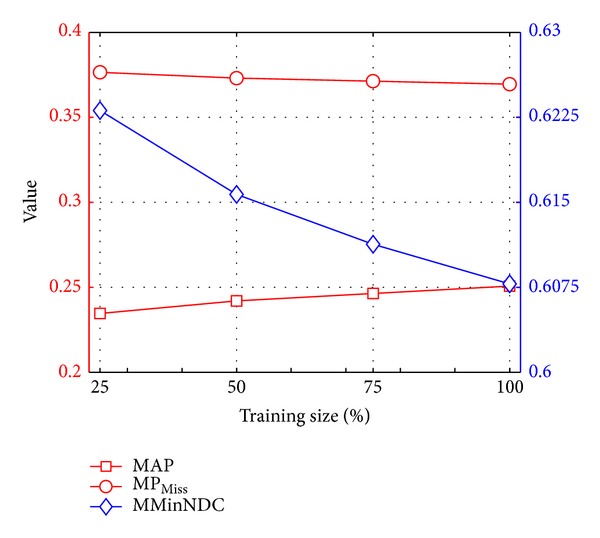
Event classification performance with respect to the training size.

We investigate the robustness of our 1000OBK event descriptor with respect to the training size of object detectors in this experiment. We train the 1000 object models by using multiple sizes of training examples, ranging from 25%, 50%, and 75% to 100% of the full ImageNet challenge 2012 training data, where we chose 100 positive images and 400 negative images as the full training data for each object category. It can be seen from the Figure 7 that there is a moderate drop for MMinNDC or MP_Miss_ and a slight increase for MAP, when the number of training size decreases from 100% to 25%. Note that a higher MAP indicates a better performance and a lower MMinNDC or MP_Miss_ indicates a better performance. The results suggested that the event classification performance dropped slightly when the training size decreased tremendously. Thus, the 1000OBK is a robust event descriptor with respect to the training size.

#### 6.5.2. Varying the Bank Size

**Figure 8:**
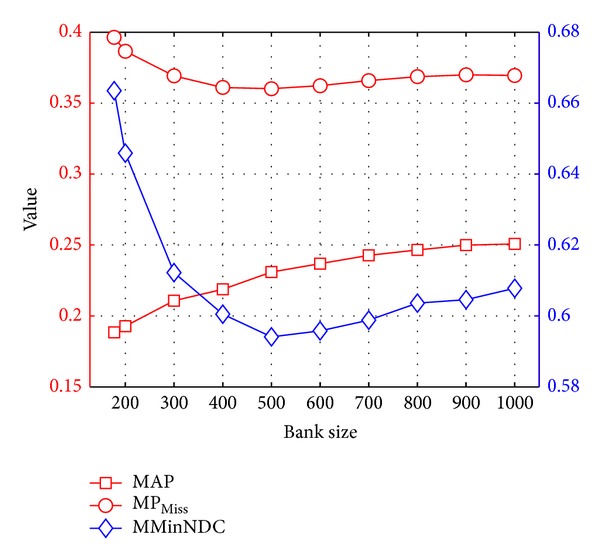
Event classification performance with respect to the bank size.

One of the major contributions in this work is the extension of the object bank [8, 9] to adapt to different event classification tasks. However, it is not given that a larger bank size improves always the event classification performance. The curse of dimensionality may prevent this intuition. In order to evaluate the impact of bank size on MED results, we conducted an experiment that varied the size of the bank from 177 detectors which constituted the original object bank [8, 9] to the 1000 detectors which constituted our 1000OBK. For each different size *k*, we chose randomly *k* detectors from the 1000 trained models to form a new *k*-OBK, where 177 ≤ *k* ≤ 1000. Then, we perform a full leave-one-out cross-validation on the TRECVID MED 2012 dataset. The results are shown in Figure 8. As we expected, the bigger bank size does indeed performed better. Specifically, we are able to achieve the MED results of MoSIFT [31] with a bank size of 500, the MED results of KLT trajectories [46, 47] with a bank size of 200, and the MED results of object bank [8, 9] with a bank size of 177, respectively.

Why could we improve the MED results a lot by varying the bank size from 177 to 1000? One of the reasons is that a given event is described by a mixture of scores from different object detectors and a spatial relationship of these objects. These events involve usually many different kinds of object categories. Furthermore, as a nongroup-sparsity classifier, the *χ* ^2^-SVM requires using more nonzero weights of objects for distinguishing different types of events. With this evidence, it is not surprising to notice the improvement of event classification performance with a larger bank size.

## 7. Conclusion

In this paper, we provided solutions to two key issues, which are a secure access control issue and a large scale robust representation issue, for a more generic and complicated online multimedia event detection. For the first issue, the paper proposed a tree proxy-based and service-oriented access control (TPSAC) model. For the second issue, the paper proposed a 1000-object-bank (1000OBK) event descriptor, a robust method for carrying out high-level event recognition on the TRECVID MED 2012 dataset. The 1000OBK leverages on the fact that a large number of object detectors, when pooled appropriately, can provide high-level semantically rich features that are superior to low-level features for MED. We also performed several experiments to demonstrate the effectiveness of our approaches for the online MED. In the future, we will use the 1000OBK representation in other useful vision applications, such as action recognition.

## Figures and Tables

**Algorithm 1:**
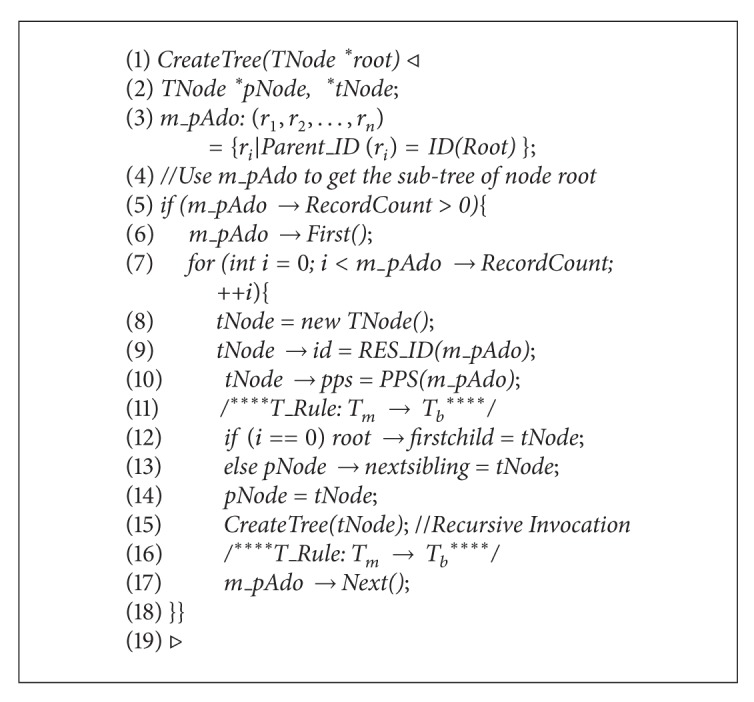
The spanning binary permission tree.

**Algorithm 2:**
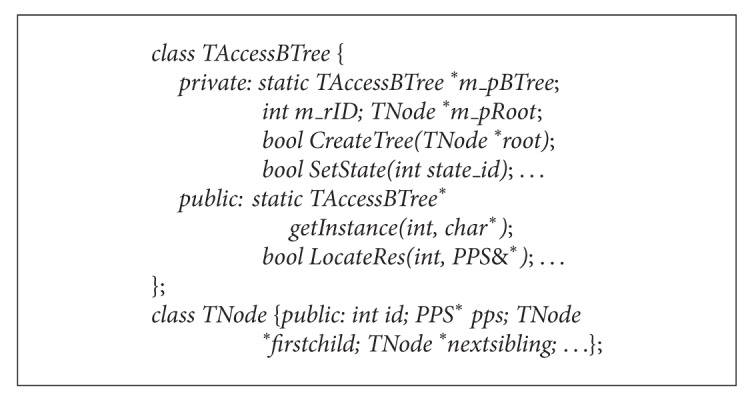
Basic classes for the TPSAC model.

**Table 1:** Comparison of different descriptors for average performance on TRECVID MED 2012 dataset.

Descriptors	MAP	MP_Miss_	MMinNDC
1000OBK	**0.2507**	**0.3695**	**0.6078**
Object bank [8, 9]	0.1884	0.3963	0.6635
MoSIFT [31]	0.2264	0.3778	0.6221
KLT trajectories [46, 47]	0.1987	0.4270	0.6944
